# Transcriptional profiling of host cell responses to encephalomyocarditis virus (EMCV)

**DOI:** 10.1186/s12985-017-0718-4

**Published:** 2017-03-04

**Authors:** Jia Wei, Haixia Zhang, Xiangrong Li, Qiongyi Li, Zhongren Ma, Jialin Bai, Zilin Qiao, Ruofei Feng

**Affiliations:** 1 The Key Bio-engineering and Technology Laboratory of SEAC, Northwest Minzu University, No. 1 Xibeixincun, Lanzhou, 730030 People’s Republic of China; 2 School of Life Science and Bioengineering, Northwest Minzu University, No. 1 Xibeixincun, Lanzhou, 730030 People’s Republic of China; 3 Animal cell Engineering & Technology Research Center of Gansu, Northwest Minzu University, No. 1 Xibeixincun, Lanzhou, 730030 People’s Republic of China

**Keywords:** Encephalomyocarditis virus (EMCV), Transcriptome analysis, Host transcriptional responses

## Abstract

**Backgroud:**

Encephalomyocarditis virus (EMCV) has been discovered on pig farms worldwide and can cause myocarditis in piglets and reproductive failure in sows. However, little is known about the host transcriptional responses to infection and host-pathogen interactions.

**Methods:**

In this study, transcription profiling was performed by Illumina RNA-Sequencing (RNA-seq) to identify EMCV induced differentially expressed genes in BHK-21 cells at serial time points (12, 24, and 30 h post infection (hpi)), using mock infected cells as control.

**Results:**

We identified 237, 241, and 207 differentially expressed genes (DEGs) respectively, majority of which were up-regulated. A large number of DEGs clustered into host defense, cellular signaling and metabolism categories. Moreover, short time series expression analysis revealed that 12 hpi was an important time point for expression change, indicating host virus resistance.

**Conclusions:**

This RNA-seq analysis provides the first data for understanding the network of virus host interactions under EMCV infection in vitro, and for identifying host components which involved in the virus infection course.

**Electronic supplementary material:**

The online version of this article (doi:10.1186/s12985-017-0718-4) contains supplementary material, which is available to authorized users.

## Backgroud

Encephalomyocarditis virus (EMCV) belongs to the *Cardiovirus* genus of the *Picornaviridae* family, and is a non-enveloped, positive single stranded RNA virus [1]. EMCV has been regarded as a worldwide distributed zoonotic pathogen infecting a broad range of host species including rodents, non-human primates, swine, as well as human beings [2–4]. Since the first outbreak in 1958, clinical outbreaks due to EMCV in swine farms have been reported in several countries [3, 5, 6]. The number of EMCV strains isolated from pigs and wild animals in China is increasing in recent years [7–9]. Although pigs are the most severely infected domestic animal species to EMCV, rodents are considered to be the natural reservoir of the virus and play a vital role in transmitting the virus to pigs [6]. EMCV induces acute myocarditis with sudden death in preweaning piglets as well as severe reproductive disorder in pregnant sows [6, 10]. Moreover, EMCV increases the risks of xenotransplantation for human recipients [11]. In rodents, EMCV induces encephalitis, member paralysis, myocarditis and type 1 diabetes [12–14]. EMCV infection was also responsible for the deaths of various animals in zoos distributed in different countries [2, 15, 16].

As the genomic RNA of EMCV is in the cytoplasm, it becomes translated into viral proteins which are indispensable for replication and viral particle formation. EMCV manipulates the host lipid metabolism and induces cell membrane rearrangement to generate replication organelles needed for replication [17]. From virus entry into host cells to release of mature viral particles, viral factors must interact with multiple cellular components of host cells and affect host transcription. However, the mechanism of interaction between host and virus is largely unknown, e.g. cellular signal transduction and host cell pathological responses triggered by EMCV infection. The initial and subsequent host responses to the virus are significant to understand host pathogen interactions and can be investigated at the transcriptional level. Next generation RNA sequencing (RNA-seq) analysis makes the study of impact by virus infection on host cell transcription at whole genome scale efficient. Previous RNA-seq transcriptomic analysis supply massive data on host transcriptional responses to various viruses. Reinhard et al. studied transcriptional host responses to feline immunodeficiency virus and discussed the similarities of that to HIV [18]. Moreover, RNA-seq analysis revealed that HCV infection affected multiple metabolic pathways in host hepatocytes [19]. Transcriptome analysis of host and virus revealed that varicella zoster virus (VSV) altered the differentiation of human keratinocyte, providing insight into the pathogenic mechanisms of VSV [20]. Melina et al. discovered that negative NF-kappa B regulators were significantly up regulated during BVDV infection by RNA-seq, suggesting a possible blocking of this signaling pathway by the virus [21].

In the present study, we investigated the host responses induced by EMCV at different time points (12, 24 and 30 hpi) using RNA-seq, and analyzed differential gene expression between uninfected and infected cell groups, as well as the dynamic of host gene expression during the virus infection. The results provide the first data for understanding of the complex mechanisms of virus host interactions during EMCV infection.

## Materials and methods

### Cells and Virus

Baby hamster kidney 21 (BHK-21) cells were maintained in Dulbecco's modified Eagle's medium (DMEM, Gibco) supplemented with 10% (v/v) fetal bovine serum (FBS, HyClone) in a humidified 5% CO2 incubator at 37 °C. EMCV PV21 strain (GenBank No. X74312) from ATCC was propagated in BHK-21 cells for viral challenge.

### Viral infection

BHK-21 cells were cultured in 75 cm^2^ flasks until they reached 80% confluency. Cells were washed with 1% phosphate buffered saline and infected with EMCV at a multiplicity of infection (MOI) of 0.01. After incubating for 1 h at 37 °C, the supernatant was removed and DMEM medium supplemented with 3% FBS was added into each flask and incubated at 37 °C. Control cells were mock infected with FBS free DMEM and treated in the same way in parallel, and then were harvested (designated as 0 hpi in dynamic gene expression analysis during EMCV infection). Subsequently, cells were harvested at 12, 24 and 30 h after EMCV infection for transcriptomic analysis. Three individual replicates were set up for each time point.

### Library construction and illumina sequencing

Total RNA was extracted from uninfected and infected BHK-21 cells using TRIzol Reagent (Life technologies, USA) according to the manufacturer's instructions. The quality of RNA was checked by capillary electrophoresis separation on Agilent 2100 Bioanalyzer (Agilent, USA). RNA samples were stored at −80 °C until further use. 1 μg of total RNA (three replicates of each time point were pooled together) were used to generate cDNA libraries. mRNA was enriched by removing rRNAs from the total RNA after DNase I treatment, by which long non-coding RNAs (used in further study) were not depleted. mRNA was chemically fragmented into 140–160 bp fragments and used as templates to synthesize cDNA by priming with random hexamers. The synthesized cDNA fragments were purified and resolved with elution buffer for end reparation as well as adding single nucleotide A (adenine). The cDNA fragments were subsequently connected with sequencing adapters. Suitable fragments were selected by agarose gel electrophoresis as templates of RCR amplification. The quantity and quality of the cDNA libraries were assessed by Agilent 2100 Bioanalyzer and ABI StepOnePlus Real-Time PCR System. Finally, the prepared libraries were sequenced using Illumina HiSeq™ 4000. The RNA-Seq raw data files have been uploaded in NCBI (http://www.ncbi.nlm.nih.gov/bioproject/356035) under the accession number PRJNA356035.

### RNA-seq data analysis

RNA-seq raw data was filtered using FASTQC software to exclude reads containing adapters, reads in which unknown bases were more than 10% and low quality reads. Clean reads were aligned and mapped against hamster reference genome (http://www.ncbi.nlm.nih.gov/genome/11998) and EMCV PV21 genome (GenBank: X74312.1) by using BWA. Bowtie was used to map reads to hamster gene reference. To analyze the expression abundance of genes, unique mapped gene reads were normalized by Fragments Per Kilobase of exon model per Million mapped fragments (FPKM) and log transformed using Cufflinks. DEGs were identified using Cuffdiff. Genes with a fold change of larger than 1.5 and with adjusted *P* values < 0.05 were considered as differentially expressed. The identified DEGs were subjected to further analysis, including Gene Ontology (GO) analysis, pathway enrichment based on Kyoto Encyclopedia of Genes and Genomes (KEGG) database, and gene expression dynamic analysis conducted by STEM [22].

### Real-time reverse transcription quantitative PCR (RT-qPCR) validation

RT-qPCR was carried out to verify the gene expression by RNA-seq analysis. 1 microgram total RNA from each group (mock infected cells, 12 hpi, 24 hpi, and 30 hpi) was used for reverse transcription using a PrimeScript RT reagent kit with a gDNA Eraser (TaKaRa). Equal amounts of RNA from three replicates were pooled together to subject to reverse transcription. All RT-qPCRs were performed in 10 μL reactions including 1 μL of cDNA product and 5 μL of SYBR Premix Ex TaqII (TaKaRa). The reactions were conducted in triplicate using a CFX96 real-time PCR machine (Bio-Rad, USA) following the temperature protocol: Initial denaturation 95 °C for 2 min; 39 cycles of 95 °C for 10 s, 56 °C for 20 s, 72 °C for 20 s. The expression fold-changes were calculated by the relative 2^-△△Ct^ method and the housekeeping gene ACTB was taken as a reference gene.

## Results and discussion

### Transcriptome sequencing and differential expression analysis

**Fig. 1:**
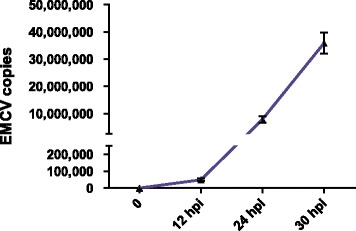
Quantification of EMCV RNA copies in infected BHK-21 cells. Numbers of EMCV RNA copies in infected BHK-21 cells at 12, 24, and 30 hpi were determined by Real-time quantitative PCR

To depict the global picture of host transcriptomic response to EMCV infection, and to identify host factors involved in the infection course, we performed RNA-seq on Illumina platform using cDNA libraries of EMCV infected and mock infected BHK-21 cells. EMCV infected cells were harvested at 12, 24, and 30 hpi. An average of 115 million reads were generated from each sample, and an average of 114 million reads passed the quality control. 72.6% of clean reads were mapped to the hamster genome in the mock infected group. In 24 hpi group, 65.3% and 7.2% of clean reads were aligned to hamster genome and EMCV genome respectively, indicating viral RNA replication before 24 hpi. In 30 hpi group, 41.5% of clean reads were mapped to EMCV genome, indicating active replication of the viral RNA (Table 1). The amounts of EMCV copies were quantified by real-time PCR, and EMCV infection was also validated. The viral RNA levels increased rapidly during infection (Fig. 1).

**Table 1:** RNA-seq reads and map rate

	Total reads	Clean reads	Hamster genome map rate	EMCV genome map rate	Total map rate
Mock	115833567	114848982	72.6%	0	72.6%
12 hpi	115104413	113918838	67.3%	0	67.3%
24 hpi	115831959	114777888	65.3%	7.2%	72.5%
30 hpi	115829611	114833476	34.1%	41.5%	75.6%

The number of DEGs (≥1.5fold, with *P* value < 0.05) at each time point was shown in Table 2. 237, 241, and 207 genes were found differentially expressed at 12, 24, and 30 hpi respectively (Additional file 1: Figure S1, Table 2). There was little difference between the total number of DEGs at each time point, however, the percentage of down regulated genes reduced drastically as the infection time grew. At 30 hpi, only 7.7% DEGs were down regulated. Even at 12 hpi, majority of the DEGs were up regulated. Moreover, DEGs with a fold change of larger than 2 at 30 hpi were much more than that at other time points (12 hpi and 24 hpi) (Table 2).

**Table 2:** Differentially expressed genes in EMCV infected BHK-21 cells at different time points

Group	DEGs (≥1.5fold)	Up (≥1.5fold)	Down (≥1.5fold)	Down/DEGs (≥1.5fold)	DEGs (≥2fold)
Mock VS 12 hpi	**237**	151	86	36.3%	47
Mock VS 24 hpi	**241**	206	35	14.5%	56
Mock VS 30 hpi	**207**	191	16	7.7%	178

*P* < 0.05

**Fig. 2:**
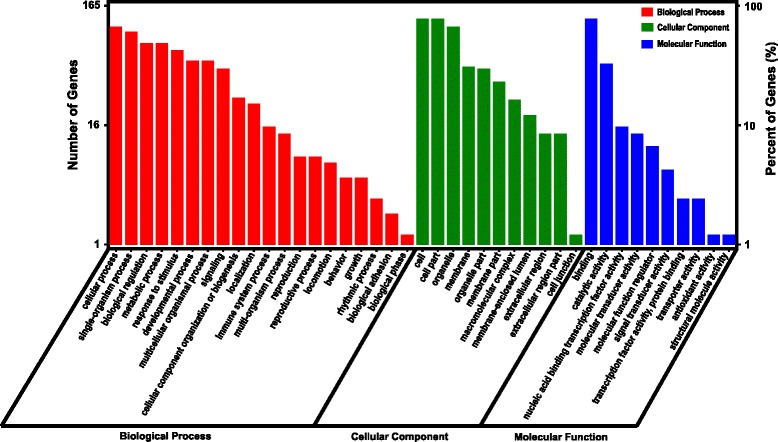
GO annotation of DEGs at 30 h after EMCV infection. The x-axis represents the functional groups, while the y-axis represents the number of genes

**Fig. 3:**
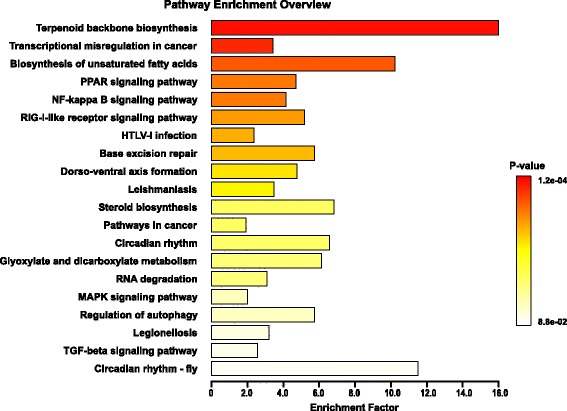
KEGG enrichment analysis of DEGs at 30 h after EMCV infection. The x-axis represents the enrichment factor, while y-axis represents enrichment KEGG pathways

DEG data were further subjected to Gene Ontology (GO) and KEGG pathway analysis. GO terms classified the functions of all differentially expressed transcripts, producing 41 functional types at 30 hpi, including immune system process, metabolic process, response to stimulus, and signaling (Fig. 2). We designated three functional categories of interest based on GO functional classification and KEGG pathway enrichment (Figs. 2 and 3): host defense, signaling and metabolism. Representative DEGs involved in host defense at the three time points, as well as the KEGG pathways which the DEGs belong to, were listed in Table 3. We identified several innate immunity pathways responsive to EMCV infection, including RIG-I-like receptor signaling pathway, TGF-beta signaling pathway, and NF-kappa B signaling pathway (Fig. 3, Table 3).

**Table 3:** Representative DEGs involved in host defenses at different time points

Mock VS 12 hpi		Mock VS 24 hpi		Mock VS 30 hpi	
Gene ID (Symbol)	Fold	Gene ID (Symbol)	Fold	Gene ID (Symbol)	Fold
101825392 (CD38)	−2.63	101824408 (NLRP3)	−3.57	101823322 (TRAF2)	2.57
101832339 (LGP2)	−2.04	101838586 (TAK1)	2.16	101824715 (TNFRSF25)	−84.75
101838791 (AP1)	2.26	101842900 (C3)	2.00	101842545 (ID3)	2.36
101842545 (ID3)	−1.96	101835832 (DCN)	67.01	101842900 (C3)	3.19
101843869 (MX2)	−2.00	101834866 (A20)	1.82	101834778 (ZFP36)	5.88
101836016 (IL18)	−1.64	101827190 (IRF7)	−1.67	101823908 (PTGS2, COX2)	4.53
101834265 (PKA)	1.67	101838791 (AP1)	1.77	101838586 (TAK1)	2.98
101834866 (A20)	−1.82	101837879 (CARD6)	1.61	101835832 (DCN)	120.55
101834601 (TGFB1)	1.62	101830419 (PI3KI)	2.45	101834866 (A20)	2.30
				101832093 (ATG12)	2.47
				101830419 (PI3KI)	3.15
KEGG pathways:					
RIG-I-like receptor signaling pathway: LGP2, TAK1, IRF7, TRAF2, ATG12					
Apoptosis: PKA, TRAF2					
Epstein-Barr virus infection: CD38, AP1, TAK1, A20, TRAF2					
TGF-beta signaling pathway: ID3, DCN, TGFB1					
Influenza A: MX2, IL18, NLRP3, IRF7, AP1					
NF-kappa B signaling pathway: A20, TAK1, TRAF2, PTGS2					
Herpes simplex infection: C3, IRF7, TRAF2, TAK1					
Cytokine-cytokine receptor interaction: TNFRSF25, IL18					
HTLV-I infection: ZFP36					
Complement and coagulation cascades: PI3KI, C3					
NOD-like receptor signaling pathway: CARD6, TAK1, NLRP3, A20					

*P* < 0.05

LGP2, which is demonstrated to coordinate with MDA5 [23], showed a fold decrease at 12 h after EMCV infection. Genomic RNA of EMCV is considered to recognized by cellular RLR receptor MDA5, not RIG-I [24]. Mx dynamin-like GTPases have been shown to have antiviral activity in type I and type III interferon systems. Recent data revealed that human MX2 served as a restriction factor for HIV-1 and other primate lentiviruses [25]. Here we showed that MX2 was down-regulated in EMCV infected BHK-21 cells at 12 hpi. The above results indicated a quick restriction of host antiviral activity by EMCV. On the other hand, antiviral genes, TAK1 and complement C3, were found up-regulated in this study at 24 hpi and 30 hpi, suggesting host defense responses. Tumor necrosis factor receptor superfamily, member 25 (TNFRSF25) transcript was discovered decreased remarkably at 30 hpi. The same TNFRSF25 was also found down-regulated in Japanese encephalitis virus infected mouse spleens [26]. Recent data indicated that knockdown of TNFRSF25 led to an increased anti-apoptotic potential [27]. EMCV is a lytic virus causing necrotic cell death, which counteracts host cell antiviral immunity by inhibiting apoptosis. Down-regulation of TNFRSF25 might contribute to this inhibition. TNFAIP3 (A20) transcript expression was found increased at 24 hpi and 30 hpi, which functions in negative regulation of NF-kappa B signaling (Table 3). A20 was demonstrated to inhibit TNF induced apoptosis [28]. Furthermore, a recent study showed that deficiency of A20 in myeloid cells improved the resistance against influenza infection, by an enhanced innate response [29]. Thus, overexpression of A20 probably counteracted the innate immune responses induced by EMCV infection, however, still needs further investigation. These findings indicated that EMCV also restricted host defense at later infection time points.

Representative DEGs involved in signaling and the KEGG pathways which the DEGs belong to, were listed in Additional file 2: Table S1. Representative DEGs involved in metabolism as well as the KEGG pathways which the DEGs belong to, were listed in Additional file 3: Table S2. Several signaling pathways, including MAPK signaling pathway, PPAR signaling pathway, mTOR signaling pathway, were widely affected subsequent to EMCV infection (Additional file 2: Table S1, Fig. 3). The differential analysis also showed that multiple metabolism pathways, especially lipid metabolism were responsive to EMCV, including Terpenoid backbone biosynthesis, Steroid biosynthesis, Biosynthesis of unsaturated fatty acids, and Glycerophospholipid metabolism (Additional file 3: Table S2, Fig. 3). Replication of EMCV RNA needs to modulate host cell lipid landscape [17]. Following the deep analysis of DEGs, we saw that the transcriptional level of host lipid metabolism were changed by EMCV.

### Dynamic host gene expression during EMCV infection

**Fig. 4:**
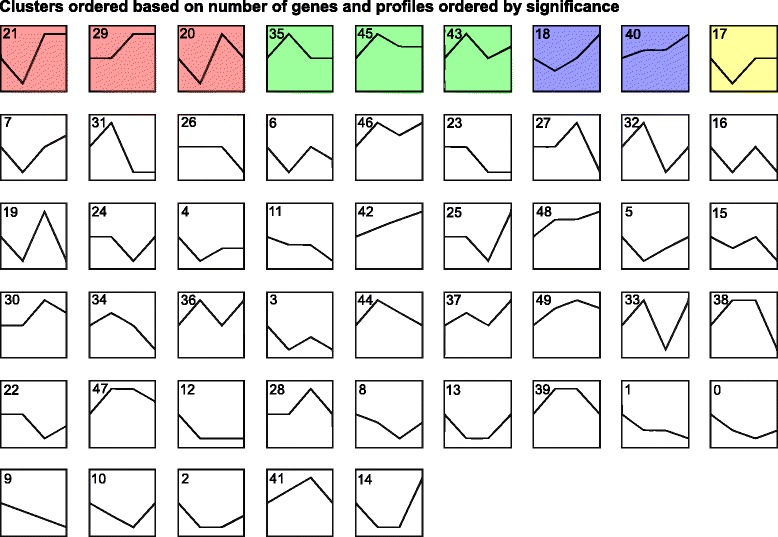
STEM analysis of short time series gene expression during EMCV infection. The number in each profile box represents the profile ID number. Statistically significant temporal expression profiles are highlighted in color (*P* < 0.05). Non-white profiles of the same color grouped together based on similarity to form a single cluster. Four time points are 0, 12, 24, and 30 h post infection

To analyze dynamic gene expression at series time points (0, 12, 24, and 30 hpi) during EMCV infection with RNA-seq DEG data, we performed pattern analysis using STEM (Short Time-series Expression Miner) software (mock infection was designated as 0 hpi). STEM is used to analyze short time series gene expression data. The results revealed four significant expression patterns (*P* < 0.05): 1. decreasing at 12 hpi and then increasing (red, including IL18, LGP2, A20 and TAK1); 2. increasing at 12 hpi and then decreasing (green, including WNT5A); 3. gradually increasing, part of the genes decreasing at 12 hpi, and then increasing gradually at later time points after infection (blue, including PLA2G2A, TXNIP, C3, TRAF2, PTGS2, CLK1, and ZFP36); 4. decreasing at 12 hpi and then increasing, but back to expression level at 0 hpi (yellow, including MRGPRG) (Additional file 4: Table S3, Fig. 4). The STEM analysis was also supported by real-time PCR later. Several antiviral genes, including LGP2, TAK1, ZFP36 and C3 decreased at 12 hpi, then increased, of which C3 increased gradually at later time after 12 hpi. These results suggested that 12 hpi was an important time point for expression pattern change, probably indicating antagonism between virus and host. EMCV might immediately reduce host antiviral gene expression to restrict antiviral activity, however, the host cells were able to make a response later to counteract with or defeat the restriction activity induced by EMCV, although were not capable of inhibiting the replication of the virus.

### Real-time PCR verification of differential expressions

**Fig. 5:**
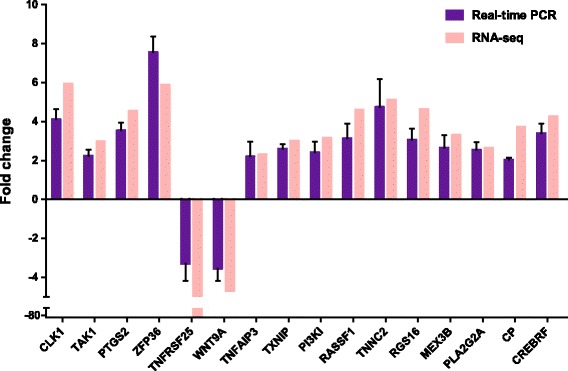
Relative Quantification of DEGs by real-time PCR for verification. Real-time PCR relative expression levels of selected genes (*purple*) at 30 hpi were compared with RNA-seq data (*pink*) at the same time point. The x-axis represents DEGs, while y-axis represents normalized fold changes of transcripts

Eighteen genes from the three functional categories were selected for validation of our RNA-seq DEG data by Real-time RT-qPCR (Additional file 5: Table S4). Gene expression profiles of Real-time RT-qPCR were compared with those of RNA-seq. Among the 18 genes, 16 genes exhibited similar expression patterns as compared with RNA-seq data. The other two genes (DDX3X and HES2) didn't have obvious expression changes based on RT-qPCR results. Fourteen of the verified differential expression genes were up regulated, and only two genes, TNFRSF25 and WNT9A down regulated. ZFP36, a RNA binding protein, which targets HIV RNA for degradation [30], showed the most significant changes in differential expression (Fig. 5). Nevertheless, the specific role of ZFP36 in EMCV infection is unclear. Moreover, short time series gene expression pattern was also verified by real-time PCR (Additional file 6: Figure S2).

## Additional files

Additional file 1: Figure S1. The Venn diagram shows common differential expressing genes at three time points (12 hpi, 24 hpi, and 30 hpi). (TIF 3137 kb)
Additional file 2: Table S1. Representative DEGs involved in signaling at different time points. (DOCX 15 kb)
Additional file 3: Table S2. Representative DEGs involved in metabolism at different time points. (DOCX 15 kb)
Additional file 4: Table S3. Representative genes in each STEM pattern (4 patterns: red, green, blue, and yellow). (DOCX 15 kb)
Additional file 5: Table S4. Selected genes for real-time PCR verification. (DOCX 13 kb)
Additional file 6: Figure S2. Real-time PCR verification of temporal host gene expression regulated by EMCV. Mock infection was designated as 0 hpi. The x-axis represents infection time, while y-axis represents normalized fold change of transcripts. (TIF 1941 kb)

## Abbreviations

DEGs: Differentially expressed genes
EMCV: Encephalomyocarditis virus
GO: Gene otology
hpi: Hours post infection
KEGG: Kyoto encyclopedia of genes and genomes
RNA-seq: RNA-sequencing
RT-qPCR: Reverse transcription quantitative PCR
