# Supplementary figures and images for: Transcriptional profiling of host cell responses to encephalomyocarditis virus (EMCV)
Source: Virol J. 2017 Mar 4;14:45. doi: 10.1186/s12985-017-0718-4 (PMC5336634; doi:10.1186/s12985-017-0718-4)

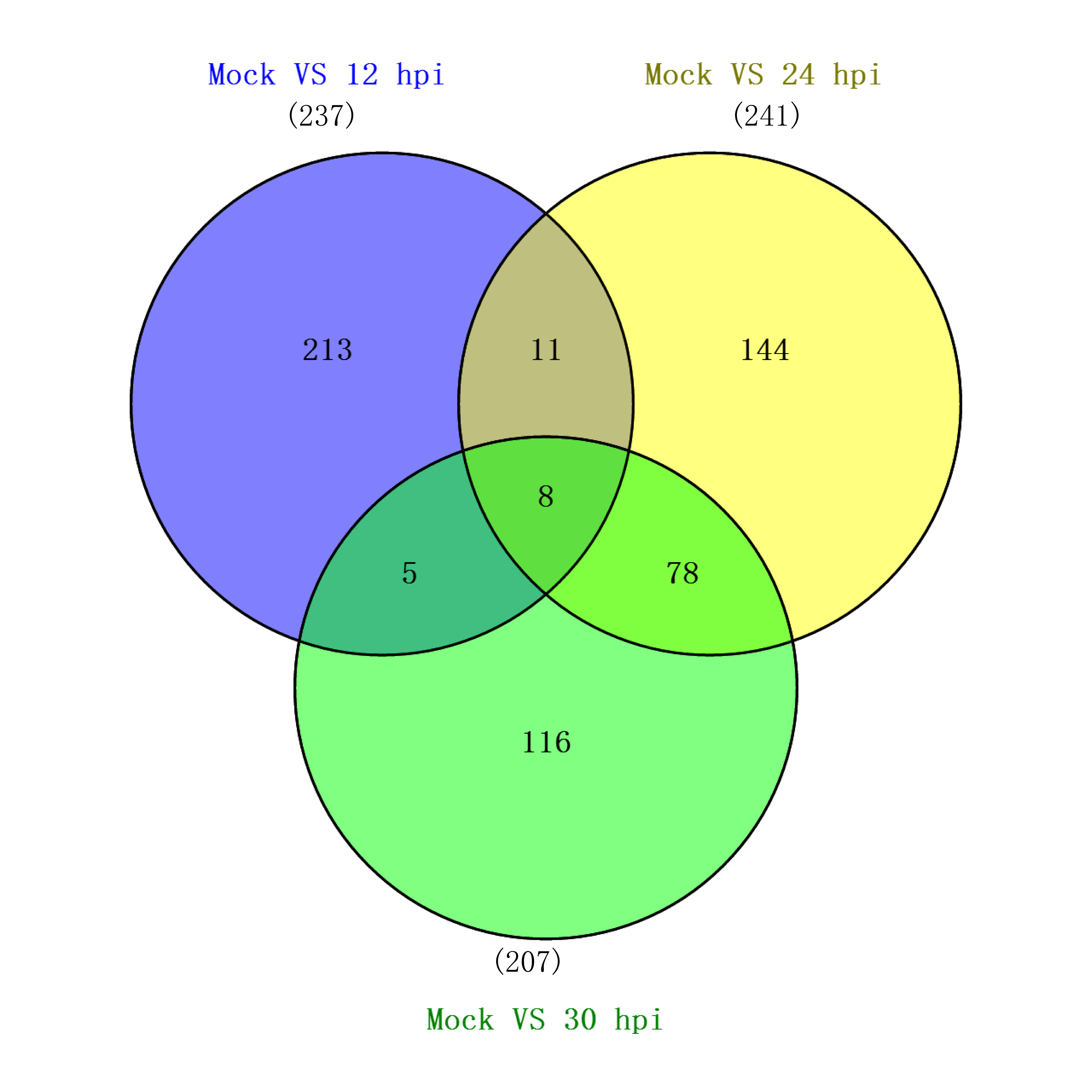

Supplement: Additional file 1: Figure S1. — The Venn diagram shows common differential expressing genes at three time points (12 hpi, 24 hpi, and 30 hpi). (TIF 3137 kb) [file 12985_2017_718_MOESM1_ESM.tif]

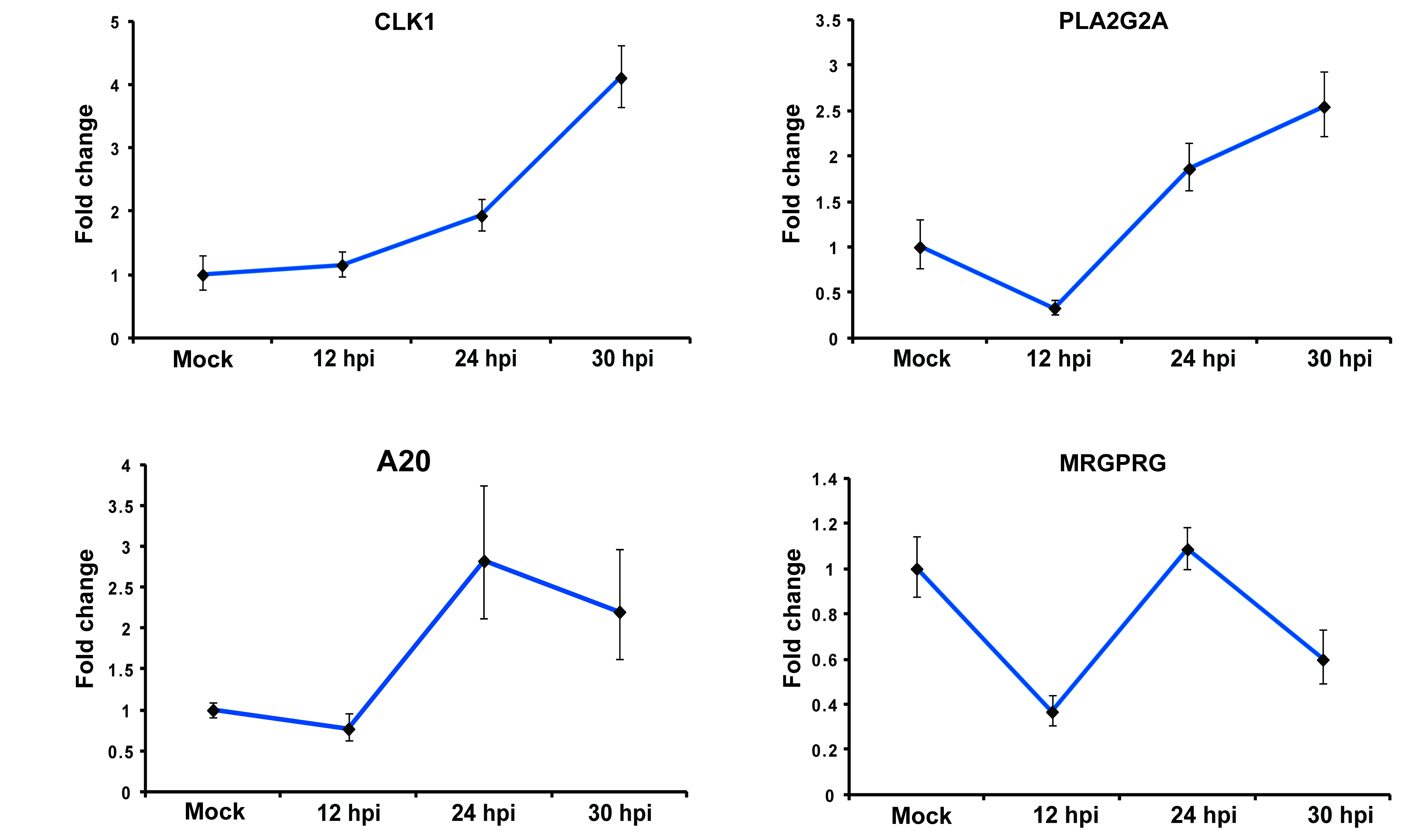

Supplement: Additional file 6: Figure S2. — Real-time PCR verification of temporal host gene expression regulated by EMCV. Mock infection was designated as 0 hpi. The x-axis represents infection time, while y-axis represents normalized fold change of transcripts. (TIF 1941 kb) [file 12985_2017_718_MOESM6_ESM.tif]
