# Supplementary material for: Transcriptional profiling of host cell responses to encephalomyocarditis virus (EMCV)
Source: Virol J. 2017 Mar 4;14:45. doi: 10.1186/s12985-017-0718-4 (PMC5336634; doi:10.1186/s12985-017-0718-4)
Supplement: Additional file 2: Table S1. — Representative DEGs involved in signaling at different time points. (DOCX 15 kb) [file 12985_2017_718_MOESM2_ESM.docx]

**Table S1** Representative DEGs involved in signaling at different time points.

| Mock VS 12 hpi | | Mock VS 24 hpi | | Mock VS 30 hpi | |
| --- | --- | --- | --- | --- | --- |
| Gene ID (Symbol) | Fold | Gene ID (Symbol) | Fold | Gene ID (Symbol) | Fold |
| 101838791 (ARHGAP33)  101831704 (SUFU)  101837262 (WNT5A)  101835885 (CYCD2)  101823630 (FGF1)  101834601 (TGFB1)  101838140 (CAB39L)  101842341 (SORBS3)  101844169 (PLEKHG2)  101844499 (TSK5)  101841845 (MRGPRG) | 2.26  2.06  1.78  1.85  -93.46  1.62  -1.82  1.52  1.73  1.59  -3.13 | 101831217 (ACSL3)  101835884 (SCD)  101839941 (HMGCS1)  101826574 (TNNC2)  101838455 (CACNA1A)  101824249 (MEGF10)  101839463 (HES1)  101842115 (FGF21)  101838791 (ARHGAP33)  101823189 (DDIT4)  101835413 (ZWINT)  101831468 (WNT9A) | 1.84  1.75  1.66  3.54  2.27  1.52  1.53  -131.58  1.77  1.55  1.58  -1.69 | 101824681 (SOWAHC)  101831217 (ACSL3)  101835884 (SCD)  101839941 (HMGCS1)  101823207 (MAP4K3)  101834246 (ATF4)  101823189 (DDIT4)  101834828 (RICTOR)  101831468 (WNT9A)  101826574 (TNNC2)  101832098 (PLCD4)  101838184 (NEDD9) | 2.15  2.31  2.05  2.66  1.92  2.51  2.09  1.98  -4.76  5.12  2.20  2.35 |
| KEGG pathways: | | | | | |
| MAPK signaling pathway: ARHGAP33, FGF1, TGFB1, CACNA1A, FGF21, MAP4K3, ATF4  Hedgehog signaling pathway: SUFU, WNT5A  Wnt signaling pathway: WNT5A, CYCD2, WNT9A  mTOR signaling pathway: CAB39L, DDIT4, ZWINT, RICTOR  PPAR signaling pathway: SORBS3, SCD, HMGCS1, SOWAHC, ACSL3  Chemokine signaling pathway: PLEKHG2, TSK5, NEDD9  Calcium signaling pathway: TNNC2, PHKA2, PLCD4  Notch signaling pathway: MEGF10, HES1 | | | | | |

*P*<0.05
