# Supplementary material for: Transcriptional profiling of host cell responses to encephalomyocarditis virus (EMCV)
Source: Virol J. 2017 Mar 4;14:45. doi: 10.1186/s12985-017-0718-4 (PMC5336634; doi:10.1186/s12985-017-0718-4)
Supplement: Additional file 3: Table S2. — Representative DEGs involved in metabolism at different time points. (DOCX 15 kb) [file 12985_2017_718_MOESM3_ESM.docx]

**Table S2** Representative DEGs involved in metabolism at different time points.

| Mock VS 12 hpi | | Mock VS 24 hpi | | Mock VS 30 hpi | |
| --- | --- | --- | --- | --- | --- |
| Gene ID (Symbol) | Fold | Gene ID (Symbol) | Fold | Gene ID (Symbol) | Fold |
| 101822505 (TNRC18)  101829014 (SETD1B)  101844757 (SUV39H2)  101828046 (PLA2G2A)  101844552 (PPAP2B)  101825925 (PDE4B)  101841090 (AMPD3)  101823324 (PI4K2A)  101826869 (GALNT5)  101828923 (ST3GAL1) | 1.95  1.95  -2.44  -2.38  -1.82  -1.75  -1.79  1.74  -1.79  1.73 | 101835628 (LOC101835628)  101843593 (MVD)  101838302 (FAXDC2)  101836410 (LPIN1)  101841994 (LPL)  101828046 (PLA2G2A)  101831418 (AIM1L)  101843896 (DDC)  101832133 (BRICD5) | 2.09  1.77  2.01  1.95  1.94  1.54  -1.82  2.27  2.62 | 101824164 (IDI1)  101843185 (HMGCR)  101843593 (MVD)  101823983 (SC5D)  101837970 (MSMOL)  101832133 (BRICD5)  101839993 (IREB2)  101828046 (PLA2G2A)  101836410 (LPIN1)  101832098 (PLCD4) | 2.00  2.25  2.48  2.37  2.10  4.33  2.13  2.63  3.48  2.20 |
| KEGG pathways: | | | | | |
| Lysine degradation: TNRC18, SETD1B, SUV39H2, AIM1L  Glycerophospholipid metabolism: PLA2G2A, PPAP2B  Purine metabolism: PDE4B, AMPD3  Inositol phosphate metabolism: PI4K2A, PLCD4  Mucin type O-Glycan biosynthesis: GALNT5, ST3GAL1  Biosynthesis of unsaturated fatty acids: LOC101835628  Terpenoid backbone biosynthesis: MVD, IDI1, HMGCR, MVD,  Steroid biosynthesis: FAXDC2, SC5D, MSMOL,  Glycerolipid metabolism: LPIN1, LPL  Histidine metabolism: DDC  Glyoxylate and dicarboxylate metabolism: BRICD5, IREB2 | | | | | |

*P*<0.05
