# Supplementary material for: Transcriptional profiling of host cell responses to encephalomyocarditis virus (EMCV)
Source: Virol J. 2017 Mar 4;14:45. doi: 10.1186/s12985-017-0718-4 (PMC5336634; doi:10.1186/s12985-017-0718-4)
Supplement: Additional file 4: Table S3. — Representative genes in each STEM pattern (4 patterns: red, green, blue, and yellow). (DOCX 15 kb) [file 12985_2017_718_MOESM4_ESM.docx]

**Table S3.** Representative genes in each STEM pattern (4 patterns: red, green, blue, and yellow)

| Patten 1 (Red) | Patten 2 (Green) | | Patten 3 (Blue) | Patten 4 (Yellow) |
| --- | --- | --- | --- | --- |
| Gene ID (Symbol) | | | | |
| 101832339 (LGP2)  101834866 (A20)  101838586 (TAK1)  101826574 (TNNT2)  101830419 (PI3KI)  101832344 (CD68)  101834002 (ABCA9)  101835125 (SRPK3)  101836016 (IL18) | | 101833091 (NCL)  101835885 (CCND2)  101837262 (WNT5A)  101834601 (TGFB1)  101824228 (MARK4)  101828419 (IRGC) | 101828634 (REL)  101842562 (TXNIP)  101842900 (C3)  101823322 (TRAF2)  101823908 (PTGS2)  101834778 (ZPF36)  101827045 (CLK1)  101828046 (PLA2G2A)  101827060 (NFKBIZ)  101823207 (MAP4K3) | 101826357 (SUMO2)  101828687 (CCDC111)  101841845 (MRGPRG)  101844060 (BRCA2) |
| Host defenses: LGP2, A20, TAK1, CD68, SRPK3, IL18, NCL, TGFB1, IRGC, TXNIP, C3, TRAF2, PTGS2,  ZPF36, CLK1, NFKBIZ, | | | | |
