# Supplementary material for: Transcriptional profiling of host cell responses to encephalomyocarditis virus (EMCV)
Source: Virol J. 2017 Mar 4;14:45. doi: 10.1186/s12985-017-0718-4 (PMC5336634; doi:10.1186/s12985-017-0718-4)
Supplement: Additional file 5: Table S4. — Selected genes for real-time PCR verification. (DOCX 13 kb) [file 12985_2017_718_MOESM5_ESM.docx]

| **Host defenses** | | **Signaling** | **Metobolism** |
| --- | --- | --- | --- |
| TNFRSF25  CLK1  DDX3X  PTGS2  TAK1  A20 | TXNIP  PI3KI  RASSF1  ZFP36  MEX3B | TNNC2  RGS16  WNT9A | HES2  PLA2G2A  CP  CREBRF |

**Table S4** Selected genes for real-time PCR verification.
